# Medical Opinion and Movement

**Published:** 1908-08-15

**Authors:** 


### Medical Opinion and Movement.

At the risk of appearing wearisome, Bier's hyperæmic method of treatment again claims attention in these columns. Professor Carl Ritter has successfully applied it in quite a novel manner for the prevention of vomiting after anæsthetic administrations. Immediately after the operation an elastic bandage is placed tightly round the neck of the patient, and kept in position for half an hour to an hour. He has employed the method after minor operations in the out-patient department where he found vomiting was especially likely to follow, owing, probably, to the lack of proper preparation, such as an in-patient receives previous to operation. In over sixty cases the procedure has been entirely successful and no vomiting has occurred. It seems, moreover, possible that a like treatment might be equally efficacious in other conditions of cerebral vomiting. Professor Ritter assumes that by the artificial hyperæmia either the brain is more easily able to rid itself of the toxic effect of the anæsthetic or is better able to resist its action. Two interesting observations appear to confirm this view. By applying the bandage before giving the anæsthetic it was found that chloroform only produced its effect very slowly. The administration lasted 10 to 15 minutes without anæsthesia, but when the bandage was removed sleep followed almost instantly. The other experiment was made on a dog severely intoxicated by subcutaneous and intramuscular injections of cocaine. Respiration was completely arrested. After all the usual means of restoration had been used without result, a bandage was tightly applied to the neck, and the animal was left for the night. On the following morning the dog was alive and well. These experiments certainly suggest that the hyperæmic method may find a new field of usefulness in cases of cerebral intoxication of all kinds.

For the past eighteen years Oppenheimer (*Munch. Med. Woch.*) has employed a form of treatment in cases of scarlatina which differs essentially from that recommended in text-books. He discards baths and wet-packs when the rise of temperature is excessive, for he dreads especially the harmful action of cold upon the kidneys. The diet at the commencement of the disease is directed towards sparing the kidneys all excessive work. Eggs, meat, and soups are forbidden, since small quantities of albumen may react unfavourably upon the kidneys. On the other hand, milk diet has no such unfavourable action, and in some 150 cases so dieted Oppenheimer has never found a case which developed nephritis. He recommends that the patient be kept in bed for six weeks. According to Heitz, however, whose cases numbered 800, milk diet does not always prevent the onset of nephritis. The appearance of such a complication would seem to vary from year to year. This observer is, however, at one with Oppenheimer about the dangers of hydrotherapy, especially at the onset of the disease, and in cases which exhibit cerebral disturbance. Later such treatment may be employed with advantage.

Glaessner contributes to the *Deutsche Medizinische Wochenschrift* a study of the results of a series of cases of local tuberculous lesions treated by means of Marmorek's anti-tubercle serum. He finds that small doses of the serum are usually beneficial, even if they cannot be regarded as curative. In two cases no benefit resulted. Where serum treatment was combined with internal medication and adequate local treatment the results were exceptionally good. Most of his cases were cases of articular or bone lesions, and in many the joints were treated by splinting and immobilisation in plaster. That with conservative treatment such cases do very well is generally admitted, and on reading the paper one is in doubt whether the good results were due to the serum injections, as the author inclines to believe, or to the accessory and strictly orthopædic treatment. In no case do the results appear to have been better than those which are obtained at Berlin by the hyperæmic treatment, which, when properly applied, has yielded astonishingly good results. The injections were given twice weekly in the form of rectal injections of 10 cc. serum, and were followed by marked improvement in the general and local condition.

In the same number Dr. Köhler gives his experience in sixty cases of tuberculous lesions also treated by means of rectal injections of Marmorek's serum. He, too, is inclined to give all the credit for the excellent results obtained in the majority of cases to the use of the serum. The pulmonary symptoms were in some cases greatly improved—the patients gained weight and complained less of cough and dyspnœa. In twenty cases, however, the lung conditions seemed to be made worse by the injections, and in twenty-six other cases the symptoms were not improved. Only in one case was the treatment followed by gradual decrease and final absence of tubercle bacilli in the sputa. In seven cases severe hæmoptysis resulted after the injections, accompanied with headaches, sweats, and dyspnœa; in three diarrhœa supervened. Köhler concludes that these results are so varied that it cannot be admitted that the serum is specific, although in some cases it certainly benefits the patient to a very great extent. Both papers are, therefore, inconclusive, and much more work is necessary before Marmorek's serum can be recommended for the general practitioner.

American surgeons have led the way in recent times in laying down definite rules of treatment for patients undergoing operative procedures, before and after operation, quite apart from the technical details of the operation itself, and it is undoubtedly by fuller attention to these matters that the best results are obtained. In a recent issue of the American *Journal of Medical Science* Dr. Cook emphasises the importance of exact estimations of blood pressure both before operation and during the time that post-operation shock persists, as a guide to the condition of the patient. He has had the blood pressure estimated at frequent intervals in a large number of cases, and as a result he draws some interesting conclusions. The blood-pressure readings must, of course, be read and interpreted in conjunction with general and other vascular symptoms, such as the condition of the heart and pulse. He finds that patients with high tension withstand the strain of ether anæsthesia and operation badly, but that if they recover from the immediate effects convalescence is generally rapid. He thinks that with hypertension previous to operation an endeavour should be made to reduce the blood pressure by doses of sodium nitrite. Low-tension cases undergo operations well, but the use of chloroform should be avoided; they appear, however, to be more liable to infection. Strychnine in large doses is indicated, and plenty of nourishing food as early as possible after the operation. Blood-pressure charts are of inestimable value as a guide to variations in the patient's condition and enable the surgeon to provide against the onset of shock or sudden cardiac dilatation. In the latter case the blood pressure may rise to 180 m.m. of mercury and vaso-dilators or even venesection are then called for.

---

The absence of any anatomical lesion underlying the condition of epilepsy has led to the view that this affection is the result of an intoxication. This hypothesis receives confirmation in the facts that injections of various substances may give rise to convulsions, and that convulsions occur in certain diseases of the excretory organs, especially of the kidneys. Voisin and Péron have shown that the urine of epileptics is hypotoxic before an attack and becomes hypertoxic after. Krainsky injected the blood of an epileptic during an attack into a dog, with the result that the animal developed convulsions to which it eventually succumbed. He believed that the toxicity of the blood consisted in excess of ammonia salts, due to a partial failure of the carbonate of ammonium to be transformed into urea. More recently Rosanoff has examined the question more closely, and following the same lines has analysed the urine of epileptics before and after the convulsive attacks. He finds that whereas in ordinary individuals the proportion of urinary ammonia to the total nitrogen content only varies at most to the extent of 0.013, epileptics show a variation amounting sometimes to 0.05. He examined altogether the urine of seven epileptics and found that with them all this proportion of ammonia to total nitrogen increased after an attack and only then. He concludes, therefore, that epilepsy is due to a peculiar disorder in the nitrogen metabolism. The prime cause and seat of such a disorder in the normal metabolic processes of the body remains still a mystery.

---

Dr. Schlecht, of Breslau, has recently adopted an ingenious method of estimating the presence of trypsine in the fæces, with a view to ascertain whether the pancreas is performing its normal functions. Müller and Kaufmann have already shown that it is possible to determine the presence of trypsine in the fæces by means of plates of coagulated blood serum. In order to make practical and clinical use of this fact Dr. Schlecht first administers the patient an enema of glycerine in the early morning. A test meal is then given, and about two hours afterwards a brisk purge. The resulting stools are triturated with glycerine, and if they are strongly acid they are rendered alkaline with a weak soda solution. Small droplets are then placed on plates of coagulated serum, and these are maintained for twenty-four hours at a temperature of 55° to 60° C. If the fæces contain trypsine a more or less definite liquefaction of the serum takes place. If trypsine is absent there is no liquefaction. By the examination of a hundred stools in this way Dr. Schlecht is convinced that this method yields reliable results. In one case, for instance, such an examination always gave a negative result, and a laparotomy showed the presence of a tumour involving the head of the pancreas and obliterating the pancreatic duct. The method is quite simple to carry out, and only requires a very small quantity of fæces, and by submitting them to the relatively high temperature it is possible to exclude to a large extent microbic impurities which might otherwise vitiate the results. By a more elaborate technique it would appear to be possible to obtain also quantitative estimations of the ferment by this method. The method certainly appears to have a clinical value in cases of obscure tumours in the pancreatic region, and may also furnish some interesting results in cases of abnormal metabolism, diabetes, etc.

---

In the *Progrès Medicale* of last week Dr. Ramond gives an interesting account of the recent visit of French physicians to the London hospitals, under the direction of M. Helme. Dr. Ramond's account is full of praise for the arrangements of our hospitals, and admires in particular the cleanliness and decorative effects of our wards, an aspect which always strikes the foreign physician who visits our institutions. The chief point of interest which he raises is the difference between our voluntary system and the system of state aid and state control exercised in France. He recognises that there is much to be said in favour of our voluntary system with its accompanying autonomous control, but points out that such a system is only possible in a country where there are possessors of large fortunes who can and are willing to make big donations. In spite of the considerable revenues which some of the hospitals possess, constant appeals are made to the public to provide for deficiencies in the revenue, and he suggests that in the event of an economic crisis of long duration the hospitals would suffer severely. While, therefore, he does not advocate the adoption of our system in France, he thinks it might be applied in a modified form. He thinks that whereas the charitable public close their purses to the Assistance Publique, they might contribute to a special appeal from individual hospitals, and he suggests that a relative autonomy should be given to each hospital by instituting a special council of administration, which would have control of all legacies and bequests. Such a council should consist of physicians and surgeons and certain influential individuals of the neighbourhood. Such views afford interesting evidence of the value that may result from international intercourse and exchange of ideas.

An interesting article on ruptures of the rectum and their treatment appears in the *Munch. Med. Wochen.* under the signature of L. Burkhardt. The author confines his attention to ruptures produced without external trauma, his observations being upon patients suffering from prolapse and rectal diverticula. In such cases rupture has been the consequence of strain, either from straining at stool or attempting to lift heavy weights. The site of injury varies greatly, but a common situation for the rupture to occur is the anterior wall of the rectum at the bottom of Douglas' pouch. Unfortunately the site is often only discoverable at the autopsy, for the rupture being nearly always intra-peritoneal, diffuse peritonitis rapidly sets in. Immediate laparotomy, though always the proper treatment, may fail to show the site of the lesion, but the author mentions a case in which a rupture caused by straining at stool gave a very exactly localised sensation. The simplest and best method of treating the tear is by direct suture; but this is often a difficult proceeding when the lesion is situated at the bottom of Douglas' pouch. Healing by first intention rarely occurs when the peritoneum and the wound have been soiled by fæcal material, however carefully the abdominal cavity be washed and cleansed. It is in most cases, therefore, better to drain the lower bowel after performing a temporary iliac colotomy. This latter can be closed when the rectum has had time to heal completely. The colotomy, moreover, has the advantage of enabling the bowels to be emptied completely, a measure which is of the utmost importance in the treatment of diffuse peritonitis.

---

That tubercle of the lung almost always begins at the apex, and generally on the right side, are facts which, although fully admitted, have never yet been satisfactorily explained. Most of the current theories proceed on the understanding that consumption arises exclusively from infection by inhalation. Thus it is stated that the largely extra-thoracic position of the pulmonary apex brings about greater inspiratory suction force there than in the rest of the lung, and likewise a weaker expiratory recoil; since the absence of bony barriers controlling the movement of the dome of the pleura causes it to precede the lower lobes in inspiration, and to lag a little behind them in expiration. This, it is argued, is a state of affairs favouring apical retention of inhaled noxæ, among them tubercle bacilli. A similar anatomical theory is that which takes note of the direction of the apical bronchioles. These certainly enter the "stem-bronchus" much more nearly at right angles than is the case lower down, and so may cause relative weakening of the apical expiratory air current. Again, as Birch-Hirschfeld has shown, the posterior sub-apical bronchus has a paravertebral and very indirect course.

---

These hypotheses, however, must fall to the ground if the universal application of the experiments of Calmette and his school, which point to absorption of tubercle bacilli from the intestinal tract as the cause of consumption, and which recent British work goes to confirm, be fully conceded. Calmette's explanation of the predilection of pulmonary tubercle for the apical region is not one of the strong points of his theory; although there is certainly the fact that miliary tuberculosis, where the infection is hæmatogenous, also appears first at the upper parts of the lungs, when it attacks those organs. The right apex is more frequently affected than the left, he says, because of its larger blood supply. The latest work bearing on this last point seems in turn to favour inhalation theories. Krönig (*Medizin. Klinink, No.* 40) describes the investigations of Helm, who made intra-tracheal injections of red lead on the bodies of new-born children and other non-tuberculous individuals, subsequently radiographing them. The pictures show that the right apical bronchial tree has much wider ramifications than the left one. This finding is in harmony with the well-known fact that the normal breath sounds at the right apex are somewhat bronchial in character. There is little room for doubt that the right apex, relatively to the left, consists largely of bronchioles, and to a slight extent only of parenchyma. However narrow may be the limits of justifiable deduction from this observation, its value cannot be disputed in view of the importance of the great subject of the pathogenesis of pulmonary tuberculosis.

---

An Italian contemporary has recently investigated the "hygiene of the ice-cream," and the results are interesting. The inquiry has rehabilitated the real ice-cream as an article of diet. When made with pure milk and cream it is stated to be a highly nourishing and easily digestible food, though its percentage of fat is comparatively small. An ordinary water ice, on the contrary, has no nutritional value whatever, and at the most can only be regarded as a depressant, and not, as usually accepted, as a stimulant. When taken before a meal it decreases the amount of gastric secretion and therefore acts as a slight inhibitant: when consumed after a meal it prolongs the period of digestion appreciably. Clinically, therefore, the water ice is to be whole-heartedly condemned, while the cream ice may be useful in many ways. When we come to the chapter on the dangers attendant on the consumption of ices, the tabulation is still more in favour of the cream ice. Impurities are to be classed as organic and inorganic, and although the former are much more dangerous they are also rarer and may almost be excluded when proper care is taken in the manufacture. The "bacillary percentage" of cream ice is higher than that of water ice: against this is the fact that the microbes in impure water ices are much more powerfully pathogenic than those in cream ice. A long list of the bacterial flora of Italian ices is given, in which B. typhosus figures prominently. Organic impurities are due not only to bad materials used in the manufacture, but to bad preservation, and to impurities in the air of the ice room. Inorganic contaminants are due to the ice chests and the apparatus. The most important inorganic impurity was found to be lead, which is present in 40 per cent. of cream ices and 69 per cent. of water ices; ammonia, sulphuric acid, and magnesium were detected in traces in all water ices.

